# Nitrous Oxide as an Emerging Cause of Subacute Combined Degeneration and Polyneuropathy: A Two-Case Report

**DOI:** 10.7759/cureus.63003

**Published:** 2024-06-23

**Authors:** Silvano Pino, Edison Vega, Marta Fragoso, Gabriel Salazar

**Affiliations:** 1 Neurology, Hospital Universitari de Terrassa, Consorci Sanitari de Terrassa, Barcelona, ESP

**Keywords:** vitamin b12, myeloneuropathy, polyneuropathy, subacute combined degeneration, nitrous oxide

## Abstract

Recreational use of nitrous oxide (N_2_O), commonly known as *laughing gas*, has increased in the last few years, bringing an increase in the number of reported cases of toxicity due to this gas. Subacute combined degeneration (SCD) of the spinal cord is the most frequently reported neurological disorder due to the use of N_2_O, as well as polyneuropathy and even psychiatric symptoms. All of these disorders are consequences of a functional deficit of vitamin B_12_. We are reporting the cases of two patients with a history of N_2_O abusive use presenting to the emergency department with progressive symptoms of paresthesia, ascending symmetric paraparesis, and gait ataxia, emulating the clinical characteristics of Guillain-Barré Syndrome (GBS). In both cases, magnetic resonance imaging (MRI) showed findings compatible with transverse myelitis of the cervical spinal cord, and electrodiagnosis studies reported the presence of polyneuropathy with a mixed mechanism. All these findings together pointed to the presence of myeloneuropathy due to a vitamin B_12_ deficit induced by the prolonged use of N_2_O. Symptoms improved gradually with vitamin B_12_ supplementation and abstinence from N_2_O. It is important to acknowledge the clinical characteristics of complications due to neurotoxicity induced by N_2_O. Such complications are potentially reversible if they are treated appropriately and quickly. Considering the increase in N2O abuse, it should be considered a probable cause when treating patients with myelopathy and/or neuropathy of an unusual etiology.

## Introduction

Nitrous oxide (N_2_O) is a colorless gas with a slightly sweet odor that is nonflammable and nontoxic. It was synthesized for the first time in 1772 by the British chemist Joseph Priestley [1]. The first use of N_2_O as an anesthetic agent was reported in 1844 by Dr. Horace Wells, a dentist who demonstrated the occurrence of insensitivity to pain during a dental extraction procedure using inhaled N_2_O [2]. Due to its anesthetic and analgesic properties, it is currently used in medicine, mainly in obstetrics and dentistry [1]. This gas has been used recreationally for many years, known as *laughing gas* for its euphoric and relaxing properties [1,3]. 

N_2_O was previously believed to be harmless until 1956 when Lassen reported a case of acute bone marrow aplasia in a patient undergoing prolonged anesthesia for the treatment of tetanus [2]. Later, in 1978, Layzer reported a total of 15 patients who developed neurological symptoms after the abusive use of large amounts of N_2_O [3,4]. Currently, it is known that the use of N_2_O is associated with the risk of developing hematological and neurological complications [2]. Its toxic effects are known to be mediated by the inactivation of vitamin B_12_ [2,5,6]. Because it is cheap and easy to obtain, its use has increased worldwide in recent years, especially during the SARS-CoV-2 pandemic, becoming the seventh most-used substance of abuse worldwide, according to the World Drug Report [1,7]. It is used in bars, nightclubs, and other entertainment facilities, particularly among the young population [1,8]. Despite its widespread use, the consequences for public health appear to be negligible because it is a relatively safe substance when consumed only occasionally [5]. Furthermore, estimates suggest that the typical user consumes a low dose of less than 10 units (cartridges) per session [5]. Despite this, the number of users who frequently consume massive quantities has recently increased [1]. This has led to a more frequent appearance among the population of some neurological complications, such as myelopathy and neuropathy [8,9]. 

N_2_O-induced myelopathy was reported for the first time in 1978 [4]. In recent years, cases of peripheral neuropathy have also been reported [9]. Furthermore, there has been an increase in the number of cases where spinal cord involvement and peripheral neuropathy coexist [3,9,10]. We report the cases of two patients with chronic use of N_2_O presenting with spinal cord involvement and polyneuropathy.

## Case presentation

Case 1

A 20-year-old male patient presented to the emergency room complaining of a three-week history of clinical symptoms initially characterized by symmetrical and ascending weakness in the lower extremities, associated with paresthesia, and progressive alteration of gait. Subsequently, he noticed weakness and paresthesia in the upper extremities, with a lack of coordination of hand movements in the last week and behavioral alteration with irritability and aggressiveness. His medical history included insomnia and anxiety treated with sertraline and quetiapine, and he had been using cocaine and cannabis since one year ago. He was brought to the emergency room one week before admission due to agitation, which was diagnosed as a possible psychotic episode due to drug use. In addition, he revealed being a habitual consumer of N_2_O through inhalation since a year ago, with an increase in frequency over the last six months, with an average of 10 units (cartridges) per day.

Neurological examination showed diminished muscle strength in the lower (3/5) and upper extremities (4/5). Decreased touch and vibratory sensations were present, and a T4 sensory level was also noted. There was a complete absence of deep muscle reflexes, and a flexor plantar cutaneous response was present bilaterally. In addition, there was dysmetria in the finger-to-nose test, as well as an increased base of sustentation and gait difficulty. Blood analysis showed a mild decrease in hemoglobin (12.5 g/dL) and red blood cells (3.81 million/mm^3^) with a mild increase in mean corpuscular volume (97 fL), with normal values of white blood cells and differential count. Toxicology tests for opiates, cannabis, cocaine, and amphetamines came back negative, as did serological tests for human immunodeficiency virus, Treponema pallidum, Hepatitis B and C viruses, Epstein-Barr virus, and cytomegalovirus. A lumbar puncture revealed the presence of elevated proteins (0.97 g/L) with albumin-cytological dissociation. Direct microbiological examination and cerebrospinal fluid culture were negative, as were polymerase chain reaction (PCR) tests for various viruses.

**Figure 1:**
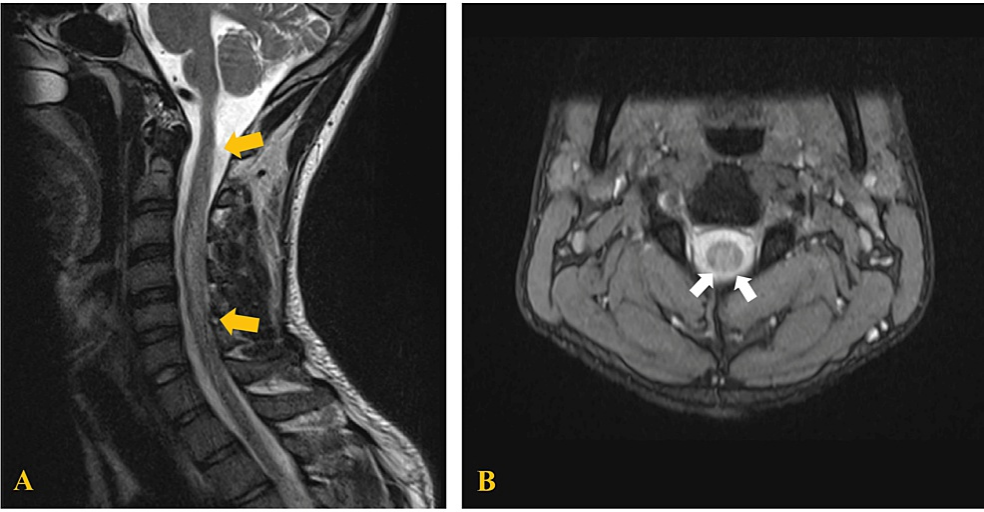
Magnetic resonance imaging (MRI) of the cervical spine of patient 1. (A) T2-weighted sagittal image of the cervical spine, highlighting the hyperintensity of dorsal columns involving C1 through C5 segments (yellow arrows). (B) T2-weighted axial image with the hyperintensity of dorsal columns (white arrow).

The electrophysiological evaluation revealed findings consistent with sensory-motor polyneuropathy, with a mixed mechanism in the upper and lower extremities. Therefore, the patient was diagnosed as having Guillain-Barré Syndrome (GBS), and treatment with intravenous immunoglobulins was started at a dose of 0.4 mg/kg/day, but the response to treatment was insufficient. So, brain and spinal cord MRIs were performed. The brain MRI showed no alterations. The MRI of the cervical spine revealed hyperintensity on T2/STIR in a wide segment of the spinal cord from C1 to C5 (Figure 1A), with involvement of more than two-thirds of the spinal cord in its transverse axis and contrast enhancement in its posterior portion (Figure 1B), as well as an increase in thickness in the medullary cord.

Given the patient's history of N_2_O consumption, the presence of neuropathy, and the addition of clinical and radiological findings consistent with myelopathy, we proposed a diagnosis of myeloneuropathy resulting from a functional deficit of vitamin B_12 _induced by chronic exposure to N_2_O. Thus, we measured vitamin B_12_ and folic acid levels, which were found to be normal (466 pg/mL and 4.04 ng/mL, respectively). However, homocysteine levels were found above the upper reference limit (19.6 µmol/L with an upper limit of 15 µmol/L).

Based on these findings, treatment with hydroxocobalamin was initiated at a dose of 1,000 µg intramuscularly once a day for two weeks, followed by once a week. He started rehabilitation sessions during his stay in the hospital and achieved partial relief of symptoms. He was discharged from the hospital three weeks after the admission and continued receiving follow-ups periodically in the ambulatory consult, exhibiting a remarkable improvement in muscle weakness and gait alteration with almost complete recovery around two months later. 

Case 2

A 21-year-old female patient was admitted with clinical symptoms for a month characterized by paresthesia that began in the lower extremities in a symmetrical and ascending manner and subsequently appeared in both hands. In addition, she developed weakness of the dorsiflexion of the left foot in the last week and postural instability and lateralization to the left, which makes it difficult for her to walk. She also reported cervical-dorsal pain. She only reported occasional alcohol consumption in her background, with no other toxic habits.

On neurological examination, muscle strength was 4/5 in the upper and lower extremities. There was decreased touch and vibratory sensation and decreased proprioception in the lower extremities. In addition, a sensory level at T5 was noted. Achilles and patellar deep muscle reflexes were abolished, and a bilateral flexor plantar cutaneous reflex was present. The Romberg sign and left lateralization in standing were also found. Blood tests showed decreased levels of hemoglobin (11.5 g/dL) and red blood cells (3.9 million/mm^3^), with normal white blood cells and a differential count. Vitamin B_12_ levels were low (165 pg/mL), and folate levels were normal (9.2 ng/mL). The serology for human immunodeficiency virus, Treponema pallidum, Hepatitis B and C viruses, Epstein-Barr virus, cytomegalovirus, and Borrelia was negative. A lumbar puncture showed no alterations. The direct microbiological examination, culture, and PCR for viruses in cerebrospinal fluid were all negative.

**Figure 2:**
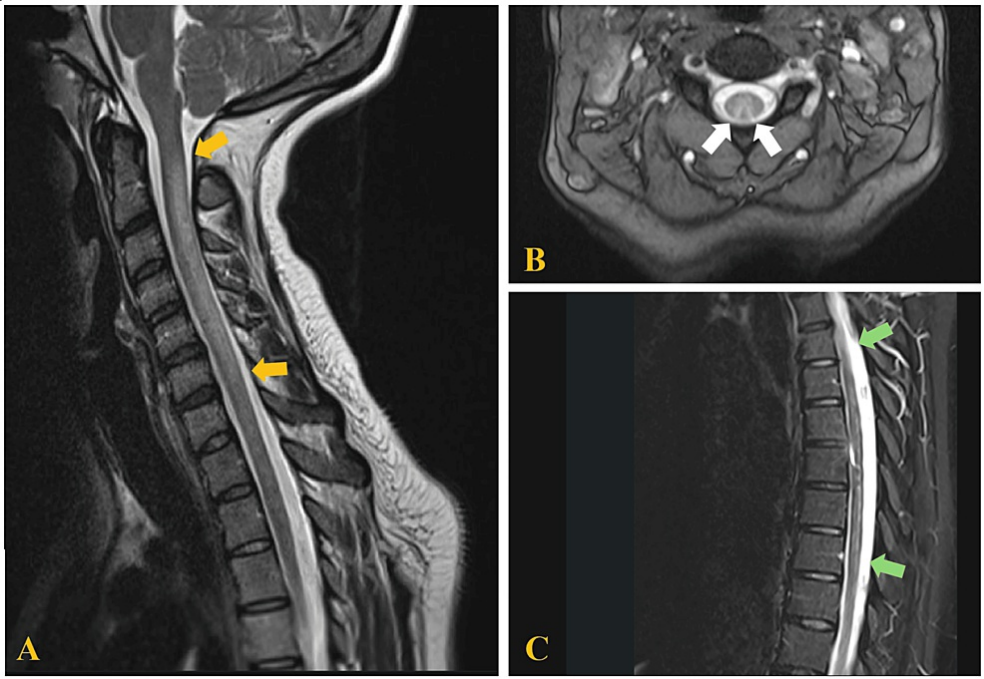
Magnetic resonance imaging (MRI) of the patient 2. (A) T2 sagittal image showing hyperintensity of dorsal columns involving segments C2 through C6 (yellow arrows). (B) T2 axial image, depicting the *inverted V* or *rabbit ears* sign (white arrows). (C) The T5–T9 segments show several foci of signal alteration (green arrows).

The electrophysiological study showed the presence of sensory-motor polyneuropathy of mixed mechanism (predominantly axonal) in both lower extremities. A brain MRI showed no abnormalities. An MRI of the cervical and dorsal spinal cord revealed a hypersignal area on T2/STIR in a wide segment of the cervical spinal cord from C2 to C6 (Figure 2A), with increased thickness involving more than two-thirds of the spinal cord in its transverse axis and with contrast enhancement in its posterior portion (Figure 2B). Additionally, some foci of signal alteration were observed in the T5-T9 segment (Figure 2C).

Considering the clinical, laboratory, neurophysiological, and imaging findings, myeloneuropathy due to an unusual etiology was highly suspected. Thus, the patient was questioned again about toxic habits, and she disclosed the chronic use of N_2_O through inhalation since a year ago, with an average consumption of six units (cartridges) per day. Based on this, we proposed the diagnosis of myeloneuropathy due to a functional B_12_ deficiency induced by N_2_O.

Treatment was initiated with methylprednisolone 1 g IV/day for 5 days, along with supplementation of vitamin B12 (hydroxocobalamin) at a dose of 1,000 µg/day for two weeks, followed by once a week. Additionally, we measured the levels of homocysteine and methylmalonic acid, and both measurements returned within normal reference values. However, it is important to say that this sample was drawn after treatment with vitamin B_12_ had been started. 

Following treatment, the patient showed partial improvement in gait, paresthesia, and limb weakness, being able to walk with no aids two weeks after the admission. She was discharged from the hospital three weeks later and continued receiving follow-ups periodically in the ambulatory consult, exhibiting a remarkable improvement in muscle weakness and gait alteration, with almost complete recovery around one month later.

## Discussion

N_2_O induces irreversible inhibition of vitamin B_12_. This is due to the oxidation of cobalt, which is part of vitamin B_12_, converting it from its monovalent active form (Co^+^) to its inactive divalent (Co^2+^) or trivalent (Co^3+^) forms [11-13]. Under normal conditions, vitamin B_12_ acts as a coenzyme for two important enzymes. In its methyl-cobalamin form, it acts as a coenzyme of methionine synthase, and in its adenosyl-cobalamin form, it participates as a coenzyme of methylmalonyl-CoA mutase [11,13].

**Figure 3:**
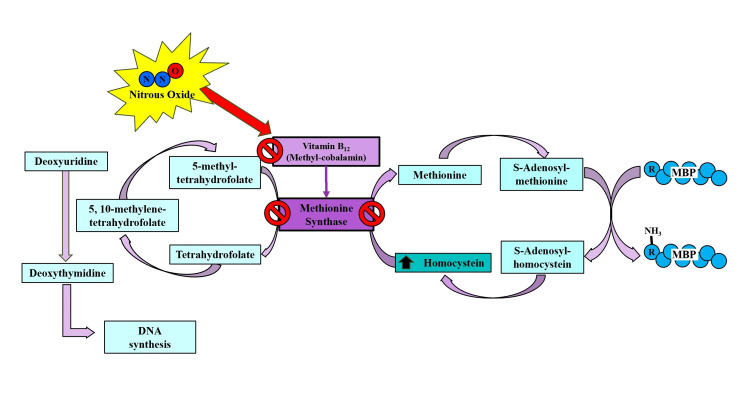
In its methyl-cobalamin biological form, vitamin B12 acts as a coenzyme for methionine synthase, which catalyzes the conversion of homocysteine to methionine as well as the conversion of 5-methyltetrahydrofolate to tetrahydrofolate. Methyl-cobalamin deficiency leads to the accumulation of homocysteine, along with a deficit in the methylation of myelin basic protein (MBP). Megaloblastic anemia can also occur due to a deficit of deoxythymidine and impaired DNA synthesis. Image credit: Author's own creation.

**Figure 4:**
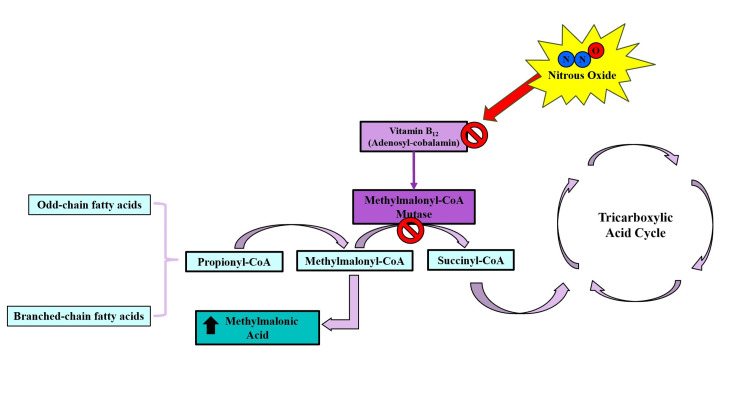
Vitamin B12, in its adenosyl-cobalamin biological form, acts as a coenzyme for the methylmalonyl-CoA mutase, which converts methylmalonyl-CoA to succinyl-CoA. A deficiency in adenosyl-cobalamin causes methylmalonyl-CoA to accumulate, directing it toward the synthesis of methylmalonic acid and the accumulation of odd-chain and branched-chain fatty acids. This modification alters the lipid composition of membranes in the nervous system. Failure to generate succinyl-CoA through this pathway also results in a deficit in energy production. Image credit: Author's own creation.

Methionine synthase changes homocysteine into methionine, which is then converted into S-adenosylmethionine, an important methyl group donor that helps methylate myelin basic protein (MBP) [14]. A highly methylated MBP is critical for maintaining myelin integrity; therefore, a deficiency in its methylation can lead to demyelination [14]. This metabolic pathway is shown in Figure 3. Methylmalonyl-CoA mutase catalyzes the conversion of methylmalonyl-CoA to succinyl-CoA. The failure of this pathway leads to the accumulation of methylmalonyl-CoA and propionyl-CoA, which retrogradely induces an increase in odd-chain and branched-chain fatty acids that accumulate in the lipids of the membranes of the nervous system. Changes in the fatty acid composition of myelin can alter its integrity and cause demyelination [5,11,13,14]. This metabolic pathway can be seen in detail in Figure 4.

The functional alteration of the aforementioned metabolic pathways leads to the accumulation and elevation of homocysteine (due to failure of methionine synthase) and methylmalonic acid (due to failure of methylmalonyl-CoA mutase) [13,14]. Furthermore, it has consequences for the function of the nervous system by generating alterations in the formation and maintenance of myelin sheaths and a deficit in energy production in the axons [1,11,13,14]. Neurological complications such as subacute combined degeneration (SCD) and polyneuropathy, previously mentioned, could result from this [6,7,11,12]. In addition, neuropsychiatric symptoms may also occur [2,10,12,14]. This may include apathy, memory impairment, personality changes, emotional lability, and, in more severe cases, impulsive and aggressive behaviors, psychosis, and visual and auditory hallucinations [10]. Although the mechanism for these symptoms is unknown, it has been proposed that an increase in the synthesis of tetrahydrobiopterin (BH4) leads to an alteration in the synthesis of monoamine neurotransmitters (dopamine, norepinephrine, and serotonin) [12].

A recent meta-analysis revealed that in patients who consume N_2_O and present with neurological complications, vitamin B12 levels may be decreased, normal, or even above normal. This variability in vitamin B12 levels results in low sensitivity (20%-50%) in the measurement of vitamin B12 [8]. Conversely, over 80% of these patients experience elevated methylmalonic acid and homocysteine levels, which may manifest before any alteration in vitamin B_12_ levels [7,8,15]. In addition, folic acid levels should be evaluated in all patients in order to identify any accompanying deficiencies [8]. Furthermore, serum copper and zinc levels can be evaluated in cases where the history of N_2_O consumption is not classic or if copper deficiency myelopathy is considered a possibility [10]. It must be considered that the severity and/or latency of clinical symptoms could depend on basal levels of vitamin B_12_ [11]. A study found that the majority of patients with normal vitamin B_12_ levels required exposure to N_2_O repeatedly and for a prolonged period to suffer neurological complications, while patients with a previous vitamin B_12_ deficiency experienced symptoms even with small amounts [11]. Therefore, the possibility of coexisting vitamin B_12_ malabsorption, which could be unmasked by the use of N_2_O, must be taken into account [8,11]. Researchers have also raised the possibility of genetic variation affecting the proteins involved in the relevant metabolic pathways, and this could predispose some individuals to adverse outcomes [3]. However, future investigations into this idea are necessary.

**Figure 5:**
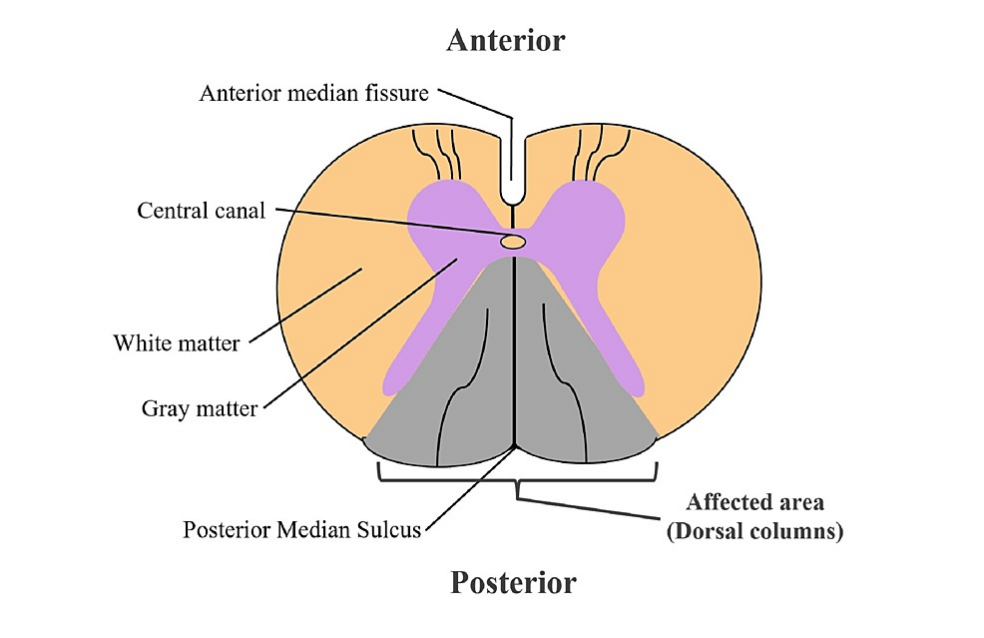
Cross-section of the spinal cord, highlighting the involvement of the posterior columns, which can occur in patients with vitamin B12 deficiency due to N2O. In addition, patients experiencing vitamin B12 deficiency from other causes, patients with copper deficiency, and those with tertiary syphilis can all exhibit this pattern. Image credit: Author's own creation.

MRI of the spinal cord can confirm the etiology and exclude other possible causes. In 50% to 100% of cases of patients with SCD, MRI shows T2/STIR signal hyperintensity in the dorsal columns of the cervical spinal cord, most frequently involving C3-C5 segments [7,16]. The axial images reveal the presence of the *inverted V* or *rabbit ears* sign [7,10,16]. This pattern of involvement is shown in Figure 5. Compared with classic SCD, N_2_O-induced SCD has a tendency to show less involvement of the thoracic spinal cord, a smaller number of involved spinal cord segments, and a higher frequency of the presence of the inverted V sign [16]. However, MRI does not categorically differentiate N_2_O-induced SCD from classic SCD or from other conditions that may share MRI findings, such as myelopathies caused by HIV, copper deficiency, vitamin E, and methotrexate toxicity [7].

Regarding neurophysiological studies in these patients, they exhibit a polyneuropathy pattern [1,2,5,6,9,11,12,17,18]. In most patients, findings are consistent with a polyneuropathy of mixed mechanism (with a predominance of the axonal component) [6,9,17]. In a recent study, Fang et al. reported that the injury rate was significantly higher in the lower limbs (72%) than in the upper limbs (42%) [9]. Considering motor nerves, most patients show reduced amplitudes, prolonged distal latencies, and slowed conduction velocities of compound motor action potentials (CMAPs) [6,9]. When sensory nerves are evaluated, the alterations in the sensory nerve action potentials (SNAPs) are predominantly a prolonged distal latency with slowed conduction velocity [9]. The type of nerve injury observed is different, depending on the upper and lower extremities. In the lower limbs, the injury tends to be higher in the motor nerves than in the sensory nerves, whereas in the upper limbs, it tends to be higher in the sensory nerves than in the motor [9].

As soon as the diagnosis of N_2_O myeloneuropathy is suspected, patients should receive appropriate treatment based on the correction of vitamin B_12_ deficiency and the suspension of N_2_O consumption. Vitamin B_12_ supplementation is achieved through the administration of vitamin B_12_ (hydroxocobalamin) at doses of 1,000 µg/day intramuscularly for two weeks, and then moving on to 1,000 µg per week until the symptoms disappear [8]. Most patients make a satisfactory recovery over a period of several weeks to a few months. Both homocysteine and methylmalonic acid levels have been reported to normalize with vitamin B_12_ replacement treatment and N_2_O withdrawal [1]. This could be a good indicator of the functional correction of vitamin B_12_. Clinical follow-up is essential to ensure a good prognosis as well as to discover other possible causes of vitamin B_12_ deficiency. In addition, patient education is needed to make them understand the toxic effects of N_2_O so that they avoid relapsing into its use.

The clinical presentation of our cases is similar to that of cases previously described by other authors [3,10,11,13,14,18,19]. They exhibit certain characteristics that need to be highlighted. First, our patients demonstrated involvement in two areas of the nervous system: the spinal cord and peripheral nerves. Therefore, the localization exercise was unique and was guided by a detailed clinical history and neurological evaluation repeated frequently, along with the performance of complementary evaluations at the appropriate time. Second, they presented with initial characteristics similar to GBS, and the discovery of albumin-cytological dissociation in one of our patients strongly suggested such a diagnosis. The co-occurrence of GBS with SCD in a patient with N_2_O consumption has previously been reported [18,19]. It is possible that an inflammatory response capable of inducing a condition similar to GBS may occur. However, a mechanism has not yet been proposed to explain its development in the context of N_2_O consumption [12]. Possible explanations for the presence of albumin-cytological dissociation include intrathecal production and release of proteins such as IgG or MBP or neuropathy-induced dysfunction of the blood-nerve barrier [20]. Third, one of our patients showed anxiety and aggressive behavior, psychiatric symptoms already described as induced by N_2_O [2,10,12,14]. However, in our patient, these symptoms did not seem to be the result of vitamin B_12_ deficiency. Fourth, a decrease in the functional activity of vitamin B_12_ was demonstrated (due to its decreased values in one patient and the elevation of homocysteine levels in the other). Therefore, rapid treatment with vitamin B_12_ supplementation was applied, leading to excellent results with almost complete recovery of symptoms in a few months. Finally, our cases are particularly outstanding in our region, since based on our review of recent publications, no case of myeloneuropathy (SCD and polyneuropathy) due to vitamin B_12 _deficiency associated with N_2_O consumption has been reported in Spain.

## Conclusions

In recent years, there has been an increase in recreational consumption of N_2_O due to its accessibility and cheapness, as well as its addictive potential. Therefore, it is important that the medical community take into account the possibility of neurological complications that can result from a deficiency of vitamin B_12_ induced by N_2_O abuse. A detailed clinical history, in association with imaging and neurophysiological studies requested at an appropriate time, will ensure a correct diagnosis. This will allow us to deliver a quick and adequate treatment through B_12_ supplementation and cessation of N_2_O consumption in order to achieve clinical resolution in our patients.
